# Co-activation of interictal epileptiform discharges localizes seizure onset zone and fluctuates with brain state

**DOI:** 10.1093/braincomms/fcaf127

**Published:** 2025-04-03

**Authors:** Samuel B Tomlinson, Benjamin C Kennedy, Eric D Marsh

**Affiliations:** Department of Neurosurgery, Perelman School of Medicine, University of Pennsylvania, Philadelphia, PA 19104, USA; Department of Neurosurgery, Perelman School of Medicine, University of Pennsylvania, Philadelphia, PA 19104, USA; Division of Neurosurgery, Children’s Hospital of Philadelphia, Philadelphia, PA 19104, USA; Division of Neurology, Children’s Hospital of Philadelphia, Philadelphia, PA 19104, USA; Departments of Neurology and Pediatrics, Perelman School of Medicine, University of Pennsylvania, Philadelphia, PA 19104, USA

**Keywords:** epilepsy surgery, interictal epileptiform discharge, intracranial EEG, network, seizure

## Abstract

Seizures are increasingly understood as emergent phenomena of complex, pathophysiologic networks. Interictal spikes are ubiquitous markers of paroxysmal synchronization in the epileptic brain and have been shown to co-activate between brain regions with millisecond-scale latencies, suggesting that they can spread through distributed networks of functionally inter-connected neuronal populations. In this study, we examined the relationship between interictal spike co-activation, seizure localization and resting-state EEG activity in children with medically refractory epilepsy. Twenty paediatric patients (mean age: 10.6 years) undergoing invasive EEG investigation with subdural electrodes were examined. Automated techniques were used to extract time-varying interictal spike co-activation networks from full-duration interictal recordings (mean: 108.6 h/patient). Networks were clustered into discrete node communities based on the conditional probability of spike co-activation. Patterns of regional and distributed interictal spike synchrony were investigated over time in relation to variables such as temporal proximity to nearest seizure and background oscillatory coherence. We demonstrate that the irritative neocortex comprises a network of semi-independent, highly cohesive communities with stereotyped local spike propagation patterns. Distributed coupling of spikes between communities was driven by outflow from the seizure onset zone and fluctuated over time in association with inter-regional coherence and temporal proximity to seizures. These results elucidate network dynamics facilitating pathologic hypersynchrony across the epileptic neocortex and further highlight the complex relationship between interictal epileptiform discharges and seizures.

## Teaser

Tomlinson *et al*. examined dynamic patterns of interictal epileptiform discharges in children with refractory epilepsy, finding that synchronous discharges arise preferentially in seizure onset nodes and propagate through networks of functionally connected brain regions.

## Graphical Abstract

**Graphical Abstract:**
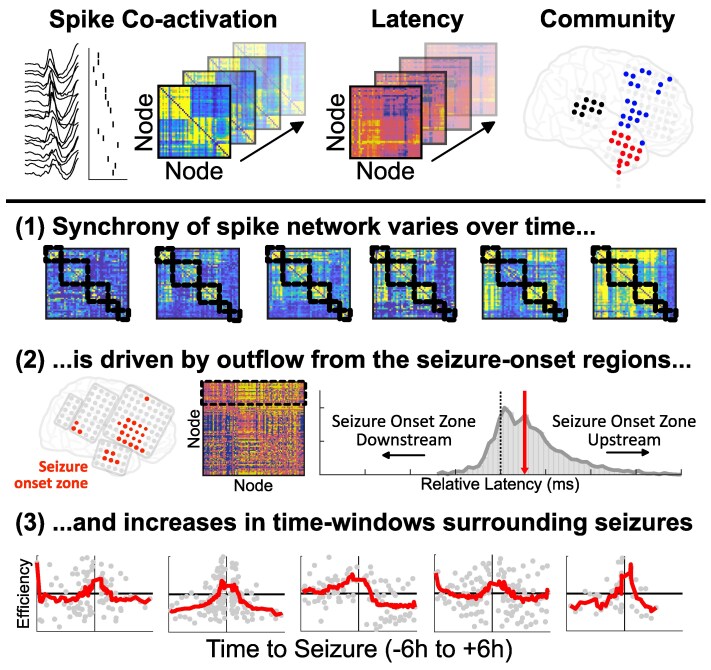

## Introduction

Interictal epileptiform discharges (IEDs), including spikes and sharp waves, are paroxysmal abnormalities recorded in the EEG of patients with epilepsy.^1^ IEDs reflect the abnormal synchronization of post-synaptic potentials generated from local ensembles of cortical neurons.^2,3^ IEDs are routinely captured during the pre-surgical evaluation and are typically interpreted as evidence of tissue hyperexcitability.^4^ However, clinicians have long recognized the perils of equating IEDs with epileptogenicity, and as such, IED analysis has a limited role in epilepsy surgery planning.

Many factors complicate the clinical interpretation of IEDs. The expanse of brain tissue exhibiting IEDs [often termed the ‘irritative zone (IZ)’] is typically broader than the seizure onset zone (SOZ), and concordance between the most active IED regions and the SOZ is inconsistent across patients.^5,6^ Complete resection of the IZ does not guarantee post-operative seizure freedom,^7,8^ and conversely, failure to resect the most active IED regions may still result in seizure cessation.^9,10^ Patients frequently exhibit multiple distinct IED populations whose relative activity rates may vary with factors such as sleep–wake cycles, medications, hormonal changes, circadian or even multi-dien rhythms.^11-14^ The frequency and distribution of IEDs vary unpredictably before seizures, creating uncertainties about their role in driving ictogenesis.^15^ Finally, IEDs have been shown to propagate across broad cortical areas, raising questions about the pathologic significance of upstream IED generators versus downstream propagation regions.^16-20^ Despite these uncertainties, IEDs remain promising biomarkers for delineation of epileptogenic tissue, given their abundance compared with seizures and their relative ease of detection using modern algorithms.

Increasingly, clinicians and researchers view the epileptic brain as a network of pathologically interrelated brain regions.^21^ From this perspective, seizures and other epileptiform paroxysms, including IEDs, result from the abnormal synchronization of network regions rather than discrete pathological zones.^22^ Various techniques to visualize epileptic network interactions have emerged, including diffusion tensor imaging, resting-state functional connectivity and direct cortical stimulation.^23^ Researchers have used network approaches to localize the SOZ,^24,25^ determine key nodes for resection^26,27^ and identify favourable surgical candidates based on the topology of network connections,^28^ among other applications.

IEDs afford a unique window through which to study epileptic networks. IEDs frequently co-occur across multiple brain regions, and their co-activation is thought to reflect underlying functional connectivity.^29^ Mapping IED co-activation enables visualization of the underlying IED network, wherein pairs of nodes (brain regions) are linked by edges (IED co-activation).^30^ Quantifying the temporal latency between coincident IEDs across nodes yields further insight into the polarity or directionality of network interactions.^31^ While characterizing epileptic networks through IED co-activation has gained traction,^32-34^ many fundamental questions remain unresolved, including how IED networks cluster into discrete node communities, how IEDs propagate through local communities and how distributed coupling of IEDs between communities varies over time in relation to factors such as seizure proximity, sleep–wake cycles and other endogenous brain states.

In this study, we investigated the phenomenon of IED co-activation in 20 children with medically refractory epilepsy. IED networks were constructed by detecting temporally coincident IEDs from subdurally implanted electrodes. These networks were then divided into communities by clustering nodes with high rates of IED co-activation. At the regional scale, IEDs tended to propagate through consistent spatiotemporal trajectories suggestive of entrained local circuits. At the global network scale, synchronization of IEDs between communities varied over time, potentially reflecting fluctuations in excitatory/inhibitory balance and oscillatory coherence. Furthermore, distributed coupling of IEDs appeared to be driven by the directional flow of IEDs from the SOZ to other functionally connected nodes. Finally, synchrony of IEDs between communities was increased in time intervals surrounding seizures, suggesting a breakdown of mechanisms that otherwise functionally isolate the SOZ from the broader network.

## Materials and methods

### Patient selection

This retrospective study comprised children with medically refractory epilepsy who underwent invasive investigation with subdural electrodes at the Children’s Hospital of Philadelphia (CHOP). The study was approved by our institutional review board. Consent was obtained in accordance with the Declaration of Helsinki. From an institutional database, 20 patients were selected on the basis of (i) at least 10 h of uncorrupted EEG data for analysis; (ii) availability of schematic electrode maps or intraoperative photographs of implant; (iii) documented presence of frequent, multifocal IED activity in the clinical report; (iv) medical record data related to patient demographics, epilepsy history and histopathologic diagnosis; and (v) at least 2 years of post-surgical seizure follow-up classified using the modified Engel scale.^35^

### Intracranial EEG acquisition

EEG recordings were acquired from subdural electrodes spaced 10 mm apart (Ad-Tech Medical Instrument Corporation, Oak Creek, WI, USA) using a Telefactor Beehive 32-128 channel Cable Telemetry Encoder digital synchronized video-EEG system (AstroNova, West Warwick, RI, USA), with a sampling rate of 200 Hz. Voltages were referenced to an electrode implanted away from the suspected epileptogenic tissue. A 60 Hz notch filter was applied.

### EEG annotation and segmentation

Recordings were independently reviewed by two expert paediatric epileptologists. Periods of excessive artefact were clipped, and electrodes with persistent artefact were discarded. For each seizure, the time of earliest electrographic change at onset and the time of seizure offset were annotated, as well as the seizure onset electrodes. The SOZ for each patient was determined via reviewing clinicians without insight into the study hypotheses. Disagreements were resolved by discussion and subsequent consensus.

Each patient’s full-duration recording was segmented into consecutive, non-overlapping, 30-min segments. For each segment, the temporal latency to the nearest seizure onset time was calculated. Ten minutes before and after each seizure were excluded to provide a peri-ictal buffer. Segments were discarded if >10 min of data were unusable due to any combination of rejected artefact, file break interruption or peri-ictal activity marked for exclusion.

### IED detection and IZ extraction

**Figure 1:**
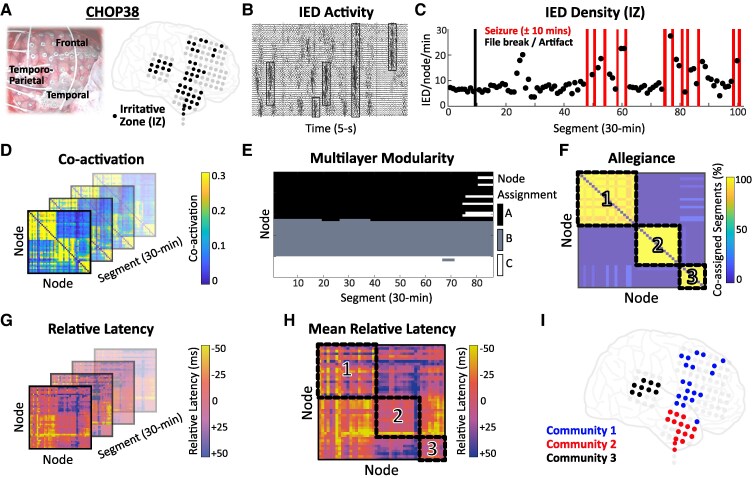
**Extraction of IED communities.** (**A**) Intraoperative photograph and schematic of electrode implant for representative patient (CHOP38). Nodes designated within the IZ are shown in black. (**B**) Representative 5-s epoch highlighting frequent synchronous IEDs (boxed). (**C**) IED density (IEDs/node/min) shown across the recording duration (total duration: 43 h, 86 segments). Each point represents the IED density for a 30-min segment. (**D**) Co-activation matrices (**C**) were computed for each segment to encode the strength of IED co-activation between node pairs. (**E**, **F**) Node subsets were assigned across segments using multilayer modularity estimation. The allegiance matrix (**A**) encoded the percentage of co-assigned segments for each node pair (range: 0–100%). (**G**, **H**) Latency matrices encoded the average relative latency (ms) between co-activated IEDs for each node pair across segments (**G**) and averaged across segments (**H**). (**I**) Louvain greedy optimization clustering of the allegiance matrix in **F** yielded a final set of node communities. IED, interictal epileptiform discharge; IZ, irritative zone.

A human-validated, automated, mimetic-based IED detector was used to identify IEDs in each 30-min segment (Fig. 1A–C). IEDs were detected based on characteristic morphological and spectral features.^36^ Patient-specific parameters including a sensitivity tuner and amplitude threshold were calibrated using representative 15-min clips. The detector outputted the electrode and peak time for each IED. The IZ was defined for each patient algorithmically by comparing the observed IED distribution (IEDs/min) to a surrogate distribution obtained by randomly reassigning IED detections to electrode labels (iterations = 1000). Electrodes with an observed IED frequency exceeding 1.5 SD above the surrogate mean were included in the IZ.^31^

### Construction of the IED network

The IED network was represented as a set of nodes (electrodes) connected by edges (IED co-activation). IED co-activation was defined as the coincident detection of IEDs between nodes within 150 ms.^29,31,37-39^ For each segment, we defined the co-activation matrix, *C*, such that *C*(*i*,*j*) encoded the conditional probability of observing a coincident IED at node *j* given the detection of an IED at node *i*, bound by 0 (no co-activations) and 1 (all IEDs at node *i* had a coincident IED at node *j*) (Fig. 1D) (Formula 1).^34^ We note that this matrix need not be symmetric. The relative latency (ms) of coincident IEDs was encoded in an inversely symmetric latency matrix, *L*, such that *L*(*i*,*j*) represented the average latency between IEDs at nodes *i* and *j*, in reference to node *i* (Fig. 1G and H).

**Formula 1.**  *C*(*i*,*j*) = *P*(*j* ∩ *i*)/*P*(*i*), where *C*(*i*,*j*) is the conditional probability of detecting coincident IEDs at nodes *i* and *j*, given detection at node *i*.

### IED community detection

Visual inspection of co-activation matrices (*C*) revealed discrete node subsets, or ‘communities’, with high rates of IED co-activation. Quantitative, unbiased extraction of node communities was achieved using multilayer modularity estimation.^40-42^ Briefly, using this technique, matrix *C* in one segment was linked to the matrix in adjacent segments, facilitating construction of a multilayer representation of the time-varying network. By maximizing a multilayer modularity quality function, each node was assigned to a community in each 30-min segment (Fig. 1E). From this, the allegiance matrix (*A*) was defined such that *A*(*i*,*j*) encoded the percentage of segments in which nodes *i* and *j* were assigned to the same community (range: 0–100%) (Fig. 1F). The Louvain greedy algorithm with an iterative resolution-optimizing routine^31,43^ was used to extract the final node communities from the allegiance matrix (Fig. 1I). This approach was found to detect a reasonable number of IED communities per patient, approximating the clinically annotated IED populations. Sparse communities comprising <5 nodes were discarded.

Basic descriptive metrics for each IED community included size (number of nodes), IED density (IEDs/node/min), and per cent overlap with the clinically defined SOZ (% nodes designated as SOZ). The consistency of community membership over time, or ‘cohesion’, was calculated as the average rate of node co-assignment across segments (range: 0–1), with 1 corresponding to a community with an identical, unvarying node membership over time.

### Propagation of IEDs within communities

**Figure 2:**
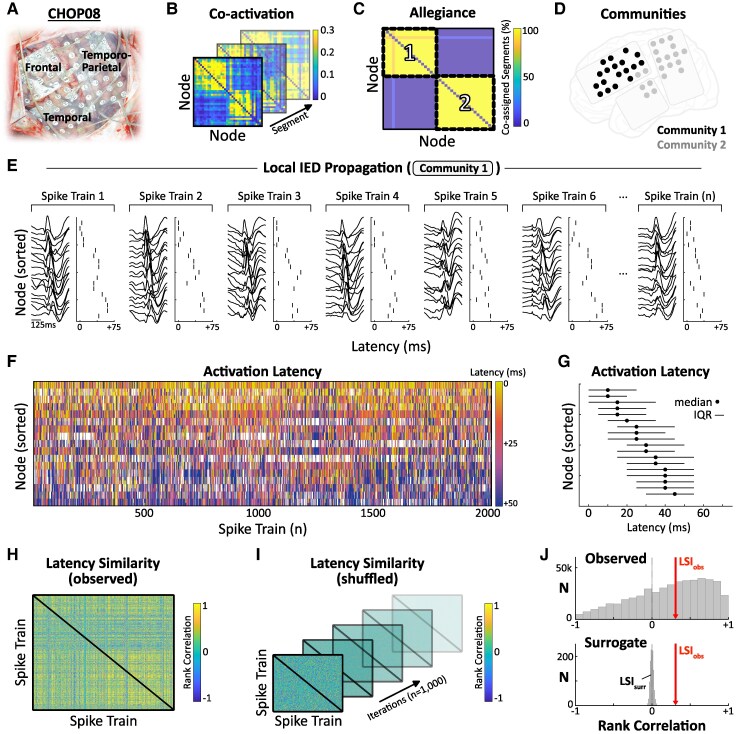
**Local propagation of IEDs within communities.** (**A**) Left fronto-temporo-parietal electrode implant for representative patient (CHOP08). (**B**) Pairwise IED co-activation computed across segments. (**C**, **D**) Clustering of the allegiance matrix revealed two distinct IED communities. (**E**) Local IED propagation for Community 1. Spike trains involving ≥50% of community nodes were extracted and rasterized based on latency (ms) from earliest spike. (**F**) Color-coded activation latencies for each spike train (*n* = 2023). (**G**) Median and IQR of activation latencies for each node across spike trains. (**H**) The rank-order consistency of spike latencies was calculated for each pair of observed spike trains using the Spearman rank correlation. (**I**) Surrogate testing was performed by shuffling each spike train and re-computing pairwise rank correlations (iterations = 1000). (**J**) The Latency Similarity Index of observed spike trains (LSI_obs_ = 0.31) exhibited <5% overlap with the surrogate distribution of LSI_shuffled_ values, indicative of a significantly non-random spike train pattern. IED, interictal epileptiform discharge; IQR, interquartile range; LSI, Latency Similarity Index.

After identifying IED communities and describing their basic composition, we next examined how IEDs spread locally within communities (Fig. 2). Propagation of IEDs through a node community can be represented as discrete spike trains, beginning with the onset electrode and proceeding in order of temporal latency (Fig. 2A–E). We aimed to characterize the consistency of spike trains across repeated events. Spike trains were extracted using a previously described technique,^31,44^ which detects multielectrode IEDs encompassing ≥50% of community nodes and sorts them according to latency from the earliest spike (Fig. 2E–G). For each pair of spike trains, we calculated the rank-order consistency of activation latencies using the Spearman correlation (Fig. 2H).^45^ The median correlation across all pairs defined the Latency Similarity Index (LSI). To assess whether the observed latency similarity (LSI_obs_) deviated from random, we performed surrogate testing by shuffling each spike train and re-calculating pairwise rank correlations, yielding a surrogate distribution of LSI_shuffled_ values (*n* = 1000) (Fig. 2I). Less than 5% overlap between LSI_obs_ and the surrogate distribution indicated a statistically non-random spike train pattern (Fig. 2J). For computational tractability, communities with abundant activity were randomly down-sampled to include a maximum of 1000 spike trains in LSI calculations.

### Dynamic coupling of IEDs between communities

**Figure 3:**
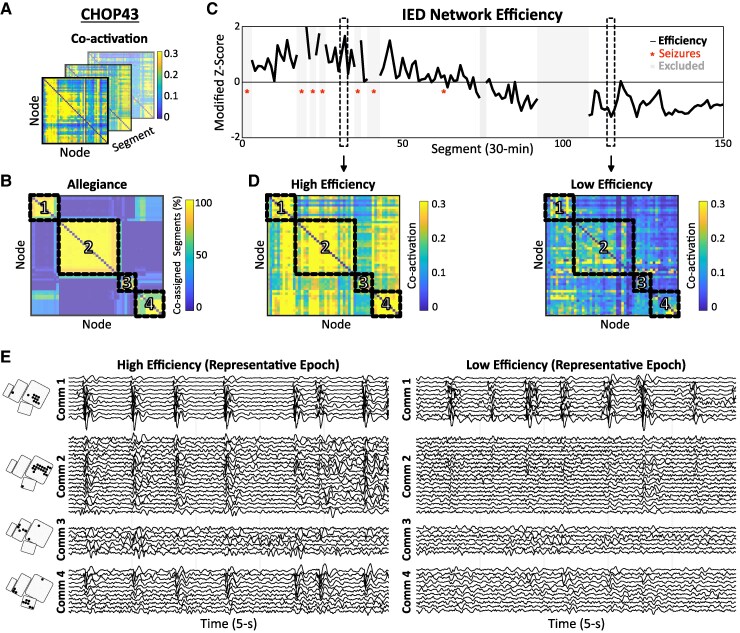
**Dynamic coupling of IEDs between communities.** (**A**) Pairwise IED co-activation computed across segments (CHOP43). (**B**) Clustering of allegiance matrix revealed four distinct IED communities. (**C**, **D**) The extent of IED synchrony across network communities was operationalized using the topologic measure of efficiency (modified *z*-score across segments). Segments of higher efficiency (D, *left*) correspond to intervals of greater IED network synchrony compared with lower efficiency segments (D, *right*). (**E**) Representative 5-s epochs clipped from high efficiency and low efficiency segments in **D**. In the high efficiency epoch, frequent runs of IEDs occurred synchronously across node communities. In the low efficiency epoch, IEDs were confined to Community 1 without propagation to other regions. Comm, community; IED, interictal epileptiform discharge.

In addition to co-activation of IEDs ‘within’ communities, we observed frequent coupling of IEDs ‘between’ communities (Fig. 3). The extent to which IEDs were synchronized between communities versus confined within local communities was quantified using the topological network measure of ‘efficiency’, which represents the average distance between node pairs, with distance inversely proportional to co-activation strength (Fig. 3A–D). Efficiency is therefore mathematically derived from the average shortest path length of the network. We note that efficiency is inversely correlated with the related network measure of ‘modularity’, which quantifies the degree to which the IED network can be clustered in a manner that maximizes the strength of within-community interactions and minimizes the strength of between-community interactions (see Supplementary Fig. 3 for elaboration). We quantified the time-varying strength of IED synchrony across the network by calculating network efficiency in each 30-min segment from the weighted, directed connectivity matrix (*C*), using the Brain Connectivity Toolbox.^46^

### IED network efficiency and seizure proximity

**Figure 4:**
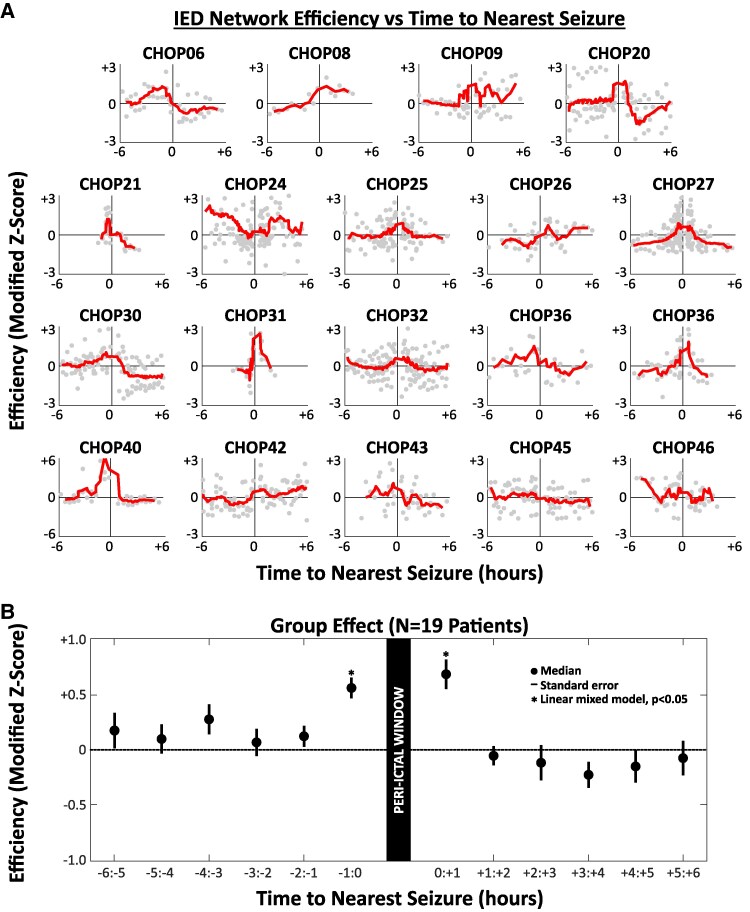
**Increased IED network efficiency surrounding seizures.** (**A**) Each plot demonstrates the relationship between efficiency (modified *z*-score) and temporal proximity to nearest seizure (hours) in the ±6-h peri-ictal interval. Data points correspond to individual 30-min segments, and red lines depict the moving average (window span = 15% of data points). Qualitative assessment reveals an increase in efficiency surrounding seizures (time point 0) for many individual patients. (**B**) For group-level quantification, time to seizure was categorized using 1-h peri-ictal bins. A linear mixed model [*n* = 19 patients; fixed effect = time to seizure (1 h bins), random effect = patient identifier] revealed significantly increased efficiency in the hour preceding and following seizures compared with the reference bin (−6:−5 h). Note that one patient (CHOP22) was excluded from this analysis due to a paucity of segments within the time window of interest. IED, interictal epileptiform discharge.

Fluctuations of IED synchrony may reflect dynamic states of greater or lesser epileptogenic network excitability. We hypothesized that increased IED synchrony occurred in time windows closer to seizures. To test this, network efficiency was calculated in each segment within ±6 h of the nearest seizure, and the modified *z*-score of each segment was computed utilizing the median absolute difference. Temporal trends were visualized at the individual-patient level and then examined at the group level using a linear mixed model assessing the relationship between efficiency and time to nearest seizure (Fig. 4). Time to seizure was categorized using 12 1-h bins spanning ±6 h to nearest seizure. We accounted for multiple observations per patient in each temporal bin by including patient identifier as a random effect in the model.

### Potential mechanisms of between-community IED synchrony

After identifying distinct node communities and demonstrating that the extent of IED synchrony throughout the network was temporally variable, we hypothesized that these relationships were generated from either global network parameters such as spectral coherence, underlying sleep physiology, or from regional shifts in excitatory/inhibitory balance. To test these possible mechanisms, we calculated spectral coherence, sleep-related spectral ratios and spike slow-wave (SW) morphology parameters, as detailed below.

### Spectral coherence

**Figure 5:**
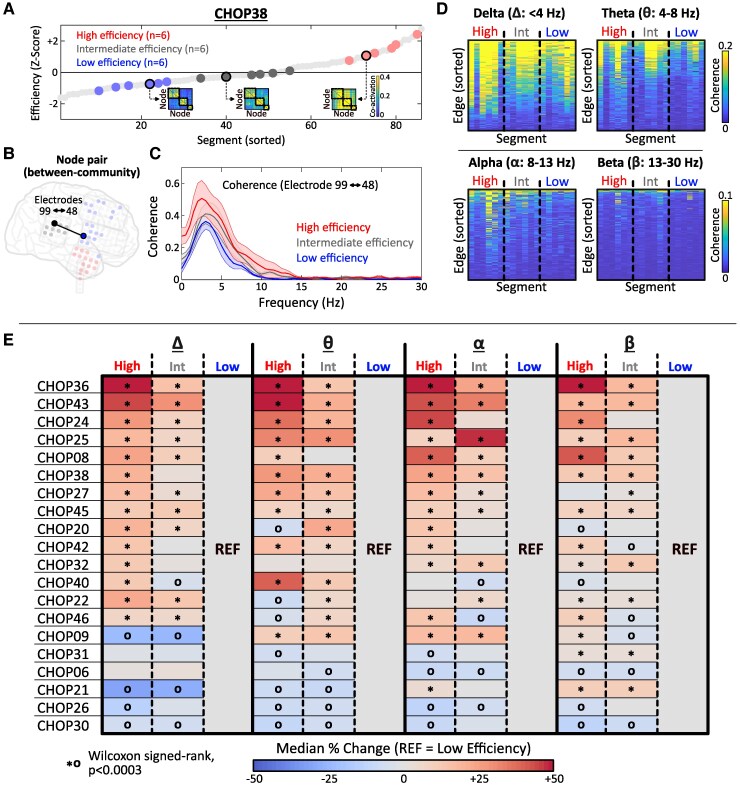
**Increased IED synchrony correlates with inter-regional coherence.** (**A**) Segments were rank-ordered based on IED network efficiency. Random selection of six ‘high efficiency’ (*top third*), ‘intermediate efficiency’ (*middle third*) and ‘low efficiency’ (*bottom third*) segments is illustrated. Insets show the co-activation matrix corresponding to representative low, intermediate and high efficiency segments (circled). (**B**, **C**) Representative example showing calculation of spectral coherence between a pair of nodes in different IED communities (line = average, shaded = standard error). (**D**) Coherence measurements for all between-community node pairs across 18 segments (6 segments per efficiency state). Plots represent results for a single patient (CHOP38). (**E**) Per cent change was calculated for each between-community node pair in reference to the low efficiency state. Matrix entries are colour coded by median per cent change from reference of all edges. Significant differences from reference are annotated (* = %change > 0, o = %change < 0, Wilcoxon signed-rank test, *P* < 0.0003). *Δ*, delta; *θ*, theta; *α*, alpha; *β*, beta.

We hypothesized that endogenous brain states, independent of IED patterns, may co-vary with the extent of IED synchrony between node communities, particularly facilitated by resting-state oscillatory activity. To investigate this, we examined the relationship between IED network efficiency and spectral coherence. For each patient, we rank-ordered segments based on efficiency and randomly selected 6 segments (3 h EEG) each from the top third (‘high efficiency’), middle third (‘intermediate efficiency’) and bottom third (‘low efficiency’) for comparison (Fig. 5A). Magnitude-squared coherence was computed in canonical frequency bands for each between-community node pair using Welch’s method (3-min random epoch comprising consecutive, 50% overlapping 1-s windows) (Fig. 5B–D). We conducted statistical analysis at the individual-patient level across frequency bands. For each node pair, the per cent change relative to the low efficiency reference state was computed, and the Wilcoxon signed-rank test was used to assess for difference [null hypothesis: %change values equal to 0; 160 planned comparisons (20 patients, 4 frequency bands, 2 comparisons/band), Bonferroni-adjusted significance threshold *α* = 0.0003] (Fig. 5E).

### Post-spike SW morphology

**Figure 6:**
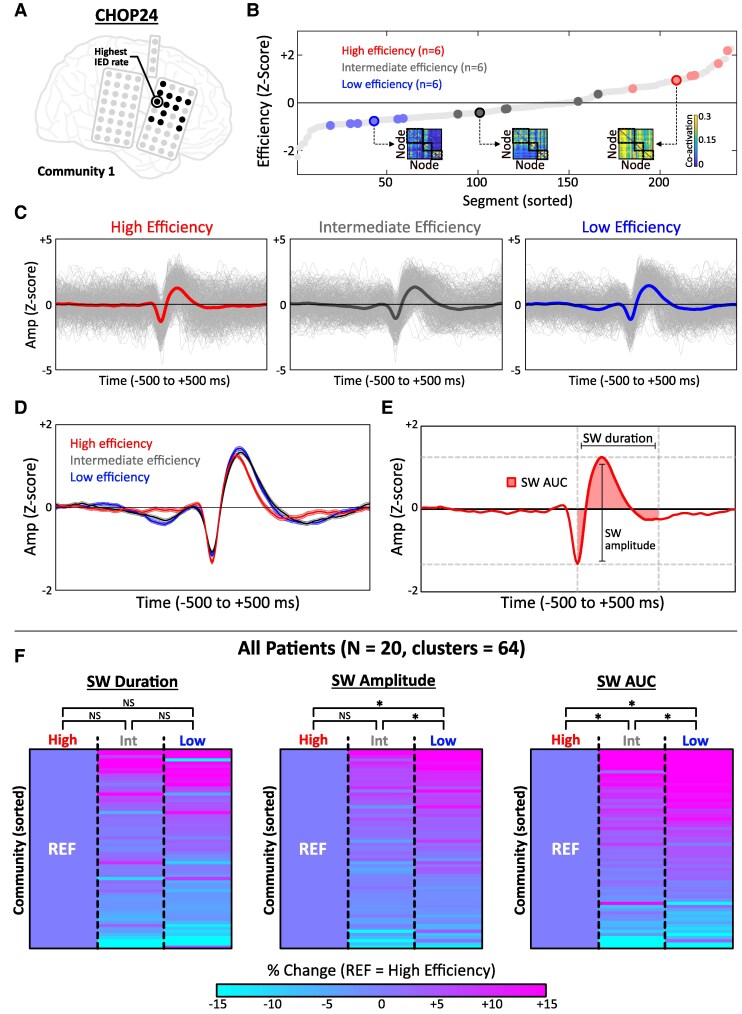
**Diminishment of post-spike SW during periods of diffuse IED synchrony.** (**A**) Electrode schematic for CHOP24. Electrode from Community 1 analysed in **C–E** is depicted. (**B**) Random selection of high, intermediate and low efficiency segments (6 segments/state). Insets show the co-activation matrix for representative low, intermediate and high efficiency segments. (**C**) Individual waveforms (grey) and grand-averaged waveforms (thick lines) are shown for IEDs at the same electrode (**A**) across efficiency states. Waveforms were randomly down-sampled to match the state with the lowest number of IEDs (in this case, low efficiency state, *n* = 385 IEDs). (**D**) Overlay of grand-averaged waveforms across efficiency states (line = average, shaded = standard error). (**E**) Three morphologic features of the post-spike SW [amplitude (*z*-scored), duration (ms), AUC] were quantified from grand-averaged waveforms. (**F**) Comparison of SW morphology across efficiency states. For clarity, per cent change values are depicted in reference to the high efficiency state. Significant differences in SW amplitude and SW-AUC were observed between efficiency states. AUC, area under the curve; IED, interictal epileptiform discharge; SW, slow wave.

We tested the hypothesis that IED synchrony was greatest during periods of excitatory/inhibitory imbalance by postulating that changes in IED waveform morphology (specifically, the post-spike slow wave) would reflect dynamic variations of inhibitory tone.^47,48^ We compared the SW amplitude, duration and integrated area during segments of high, intermediate and low IED network efficiency (Fig. 6). This analysis was performed for each node community by examining the electrode with the highest IED rate and computing the grand-average IED waveform of all detected spikes (waveforms randomly down-sampled to the smallest number of events across efficiency states; see Supplementary Table 2). Group-level differences across efficiency states were evaluated using Friedman’s test for repeated measures with *post hoc* pairwise Wilcoxon signed-rank tests [adjusted significance threshold for 9 planned comparisons, *α* = 0.005] (Fig. 6F).

### Sleep–wake state

The frequency and synchrony of IEDs have been shown to vary heterogeneously with sleep.^14,34,49,50^ Although video annotations were not available for review, we computed a simple spectral power ratio [alpha–delta power ratio (ADR)], which has been used elsewhere to approximate sleep–wake cycles (see Supplementary Fig. 4 for illustration).^12^ ADR is generally interpreted to distinguish periods of sleepfulness (low ADR) versus wakefulness (high ADR). ADR was calculated in each segment from 60 random 5-s epochs (5 min/segment) as the ratio of power in the alpha (8–13 Hz) and delta (0.2–4 Hz) frequency bands, and the relationship between IED network efficiency and ADR was examined at the individual-patient level.

### Latency of IED co-activation and SOZ localization

**Figure 7:**
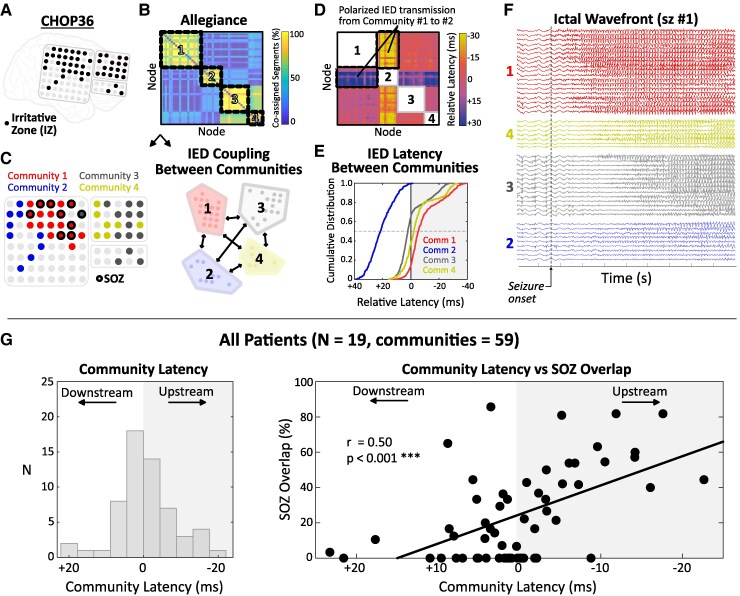
**SOZ drives coupling of IEDs between communities.** (**A**) Electrode schematic and IZ (black) for CHOP36. (**B**, **C**) Four IED node communities were extracted from the allegiance matrix. SOZ nodes (*n* = 9) are highlighted with thick circles. Community 1 (red) exhibited the strongest SOZ overlap (8/19 nodes, 42.1%). (**D**) Latency matrix demonstrating temporal relationships between communities. For example, IED co-activation between Communities 1 and 2 is asymmetric, such that IEDs occur earlier (i.e. upstream) in Community 1. (**E**) Cumulative distribution of between-community latencies, with the median defining the Community Latency (ms). (**F**) Ictal propagation wavefront (10-s) for representative seizure from CHOP36. The most upstream interictal community (#1) exhibits the earliest ictal activation and had the strongest overlap with SOZ. (**G**) Group-level analysis of Community Latency versus SOZ overlap. Communities with more negative Community Latency (i.e. upstream ‘distributors’) exhibited greater overlap with SOZ (Spearman’s *r* = 0.50, *P* < 0.001, line = least-squares fit). Each data point represents a node community (*n* = 59). IED, interictal epileptiform discharge; IZ, irritative zone; SOZ, seizure onset zone.

**Figure 8:**
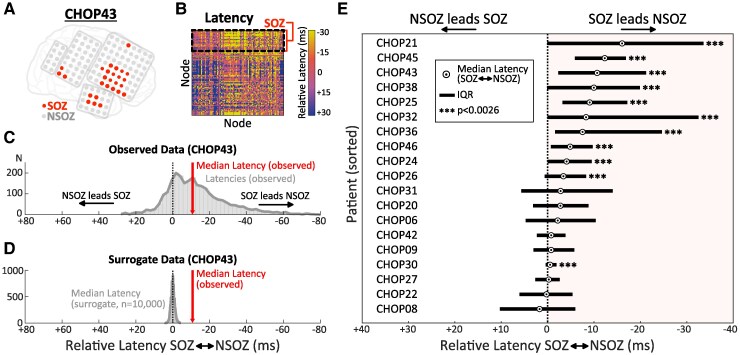
**SOZ nodes lead NSOZ nodes in coupled IEDs.** (**A**) Electrode schematic for representative patient (CHOP43) with depiction of SOZ nodes (red). (**B**) Latency matrix for all recording electrodes, with SOZ electrodes occupying the top matrix rows. (**C**) Observed distribution of IED latencies for SOZ-NSOZ interactions. The median latency (−10.7 ms) reflects a tendency for coincident IEDs to occur earlier in SOZ nodes than NSOZ nodes. (**D**) The observed median latency was significantly outside a surrogate distribution obtained by randomly shuffling SOZ identifiers (iterations = 10 000). (**E**) Box plots depicting the observed median latency (circle) and IQR of SOZ-NSOZ interactions for each patient. *P*-values represent the fractional overlap between observed median latency and the surrogate distribution (*α* = 0.0026 to adjust for 19 analysed patients). IED, interictal epileptiform discharge; IQR, interquartile range; NSOZ, non-seizure onset zone; SOZ, seizure onset zone.

Finally, we addressed the question: how does IED coupling between node communities relate to SOZ localization? Specifically, we hypothesized that communities with greater SOZ overlap would ‘lead’ (i.e. exhibit earlier relative latency) synchronous IEDs in other communities, suggesting that these discharges were originating in the SOZ and propagating to other functionally connected network regions. We defined the Community Latency as the median relative latency (ms) of all interactions with nodes in other communities, such that communities with more negative values were considered temporally upstream (Fig. 7A–E). The relationship between SOZ overlap (%) and Community Latency was examined using the Spearman rank correlation (Fig. 7G). Finally, as a straightforward assessment of IED transmission between SOZ and non-SOZ (NSOZ) nodes, we computed the median and interquartile range (IQR) of relative latencies for all SOZ–NSOZ node pairs in the recording array and compared this with a surrogate distribution obtained by randomly shuffling the SOZ identifiers (iterations = 10 000) (Fig. 8).

### Statistical analysis

Unless otherwise indicated, summary statistics are reported as mean ± standard deviation (SD). Analyses were performed using MATLAB 2023a (Natick, MA, USA), except for the mixed model assessing efficiency versus temporal latency to seizure, which was conducted in RStudio.

## Results

### Clinical characteristics

Twenty children (15 males, mean age: 10.6 years, range: 1–21 years) with medically refractory epilepsy were examined (Table 1). Some of these patients (12/20) were described in a previous publication.^31^ A variety of histopathologic diagnoses were represented, with focal cortical dysplasia (FCD) as the most common entity (14/20, 70.0%). An average of 108.6 ± 120.2 h of EEG data was analysed per patient (range: 11.5–504.0 h). On average, 104.7 ± 15.4 subdural electrodes were implanted, and 11.2 ± 6.1 electrodes were excluded due to persistent artefact. The IZ encompassed 48.1 ± 10.3% of the recording array, comprising 44.4 ± 9.0 electrodes/patient (range: 25–59 electrodes).

**Table 1:** Clinical characteristics of study cohort

ID	Gender	Age (y)	30-min Segments, *N* (hours)	Grid Coverage	Implanted (*N*)	Rejected (*N*)	IZ Nodes (*N*, %)	Histopathology	Outcome (Engel)
**CHOP06**	Female	16	51 (25.5)	R FT	92	0	25 (27.2)	Tuberous sclerosis	II
**CHOP08**	Male	18	38 (19.0)	L FTP	100	8	38 (41.3)	FCD	IV
**CHOP09**	Male	7	142 (71.0)	R FTP	104	9	40 (42.1)	FCD	IV
**CHOP20**	Male	11	142 (71.0)	L F	128	5	50 (40.7)	Neurofibromatosis	III
**CHOP21**	Male	1	42 (21.0)	L FTP	109	21	59 (67.0)	FCD	II
**CHOP22**	Male	5	168 (84.0)	R F	95	7	41 (46.6)	FCD	I
**CHOP24**	Female	15	242 (121.0)	R FP	68	10	32 (55.2)	FCD	III
**CHOP25**	Male	12	178 (89.0)	B/l O	123	20	43 (41.7)	N/A	N/A
**CHOP26**	Male	17	81 (40.5)	L TP	100	7	42 (45.2)	CVA, post-meningitis	I
**CHOP27**	Female	11	172 (86.0)	R FTP	116	12	52 (50.0)	CVA	I
**CHOP30**	Male	21	519 (259.5)	R TO	125	10	46 (40.0)	FCD	I
**CHOP31**	Male	5	23 (11.5)	L F	94	5	43 (48.3)	FCD	I
**CHOP32**	Male	12	174 (87.0)	L FTP	110	21	29 (32.6)	FCD	I
**CHOP36**	Male	14	1008 (504.0)	R F	96	16	50 (62.5)	FCD	I
**CHOP38**	Male	7	86 (43.0)	R FTP	111	12	45 (45.5)	FCD	III
**CHOP40**	Male	8	65 (32.5)	R F	115	23	53 (57.6)	FCD	IV
**CHOP42**	Female	5	341 (170.5)	R F	106	10	49 (51.0)	FCD	III
**CHOP43**	Male	16	124 (62.0)	R FTP	118	7	51 (45.9)	FCD	I
**CHOP45**	Female	3	217 (108.5)	R FT	75	8	41 (61.2)	Ganglioglioma/MTS	I
**CHOP46**	Male	8	57 (28.5)	L FTP	109	12	59 (60.8)	FCD	I

B/l, bilateral; CVA, cerebrovascular accident; F, frontal; FCD, focal cortical dysplasia; IZ, irritative zone; L, left; MTS, mesial temporal sclerosis; O, occipital; P, parietal; R, right; T, temporal.

### Basic composition of IED communities

In total, 64 IED communities were extracted across patients (range: 2–5 communities/patient) (Supplementary Table 1). Communities contained an average of 13.4 ± 5.8 nodes. The average community IED density was 6.7 ± 4.2 IEDs/node/min. The relative proportion of IEDs accounted for by each community fluctuated over time and was highly variable across patients (Supplementary Fig. 1). Community membership, however, was remarkably stable across segments (median cohesion: 0.99, IQR: 0.82–1.00, range: 0.56–1.00), indicating that redistribution of nodes between communities was uncommon (Supplementary Table 1). Nearly half of examined communities (28/64, 43.8%) demonstrated an identical node complement across all analysed segments (i.e. cohesion = 1).

### Stereotyped local IED propagation characterizes most communities

For each IED community, the sequential recruitment of nodes during propagating IEDs was evaluated by quantifying the consistency of observed spike train latencies across repeated discharges (LSI_obs_) and comparing this to shuffled surrogate data (Fig. 2). A majority of IED communities (43/64, 67.2%) exhibited a significantly non-random consistency of observed spike train latencies compared with the surrogate distribution (Supplementary Table 1). Communities with a higher frequency of spike trains exhibited higher LSI_obs_ values (Spearman’s *r* = 0.58, *P* < 0.001; Supplementary Fig. 2), suggesting that more active communities also had more spatiotemporally consistent spike trains.

### IED communities became more synchronized around seizures

We operationalized the extent of IED synchrony across network communities using the topological measure of efficiency (Fig. 3; Supplementary Fig. 3). Each patient exhibited fluctuations of network efficiency over time (e.g. Fig. 3C). When analysing efficiency in the 12-h peri-ictal interval at the individual-patient level, we observed qualitatively increased efficiency in the time windows surrounding seizures in many patients (Fig. 4A). At the group level, a linear mixed model assessing the relationship between time to seizure and network efficiency revealed increased efficiency in the 1-h window preceding seizure onset (−1:0 h, *P* = 0.043), as well as the hour following seizure offset (0:+1 h, *P* = 0.014), compared with the reference bin (−6:−5 h) (Fig. 4B). No other time windows differed significantly from the reference. We note that one patient (CHOP22) was excluded from this analysis due to a paucity (<10) of segments within the 12-h interval of interest.

### IED synchrony correlated with increased spectral coherence and change in post-spike SW morphology

We considered three resting-state factors that could relate to the extent of IED synchrony across the network: (i) sleep–wake state, (ii) changes in local cortical inhibition and (iii) global network shifts in oscillatory synchrony. For shifts in synchrony, coherence between node communities was compared across states of high, intermediate and low IED network efficiency (Fig. 5). Patient-level findings are depicted in Fig. 5D. In general, high and intermediate IED efficiency segments were associated with increased coherence between communities relative to low efficiency segments. This effect was most consistent across patients in the delta and alpha frequency bands, in which 14/20 (70.0%) patients exhibited significant increases in between-community coherence during high efficiency segments compared with the low efficiency reference.

We next analysed IED waveforms from 64 total electrodes across the 3 efficiency states and quantified morphologic features of the post-spike SW from grand-averaged waveforms (Supplementary Table 2; Fig. 6; Supplementary Fig. 5 for visualization of all grand-averaged waveforms). No significant differences were observed between efficiency states regarding SW duration: high efficiency (226.3 ± 51.3 ms), intermediate efficiency (236.5 ± 54.9 ms) and low efficiency (237.0 ± 51.8 ms), *P* = 0.47 (Friedman’s test). There were significant differences observed across efficiency states for SW amplitude (*P* = 0.0034); amplitude was significantly greater in low efficiency segments compared with intermediate efficiency (*P* < 0.001) and high efficiency (*P* < 0.001) segments (Fig. 7F, middle). Similarly, there were significant differences observed for SW area under the curve (SW-AUC) (*P* < 0.001), with *post hoc* pairwise differences observed between all pairs of efficiency states (Fig. 6F, right) in a step-wise manner such that SW-AUC was greatest in low efficiency segments.

Finally, analysis of network efficiency in relation to spectral patterns thought to index sleep–wake state (ADR^12^) is presented in Supplementary Fig. 4. We note here that the correlation between ADR and network efficiency was extremely variable, both in terms of strength and directionality, and that no consistent patterns emerged across patients.

### SOZ nodes drive IED coupling between communities

We examined co-activation latencies between node communities to assess whether synchronous IEDs reliably appeared earlier (upstream) or later (downstream) in certain network regions. One patient (CHOP40) with a generalized ictal onset pattern was excluded from this set of analyses because the SOZ incorporated all electrodes in the array. Across the remaining 19 patients (*N* communities = 59), communities with upstream co-activation latencies (i.e. more negative Community Latency) exhibited greater overlap with the SOZ (Spearman’s *r* = 0.50, *P* < 0.001; Fig. 7G). When examining IED transmission between all SOZ and NSOZ node pairs (Fig. 8), we observed polarization favouring SOZ leading NSOZ in 17/19 patients, which was significant upon surrogate testing in 11/19 patients (57.9%). No patient exhibited a statistically significant tendency for NSOZ nodes to lead SOZ nodes (Fig. 8D).

## Discussion

Although IEDs are an electrographic hallmark of epilepsy, the precise pathophysiological significance of IEDs has been difficult to define.^4^ In this study, we examined IEDs from the perspective of an effective connectivity network, wherein brain regions were linked by their tendency to co-activate during IEDs, and directionality was inferred from the millisecond-scale lags between IEDs. We found that the epileptic brain segregated into discrete node communities defined by high rates of IED co-activation, stable community membership over time and stereotyped local propagation patterns in most communities. Furthermore, we found that coupling of IEDs between communities fluctuated over time and correlated with seizure proximity, coherence and excitatory/inhibitory balance. Finally, we replicated prior studies demonstrating that transmission of IEDs between SOZ and NSOZ regions is polarized favouring outflow from the SOZ to other functionally connected but presumably less pathologic network nodes.^16,32,34,39,51,52^

Several potential clinical implications emerge from this set of observations. First, patients routinely exhibit multiple independent or semi-independent IED populations, and clinicians have few tools at their disposal when appraising the relative epileptogenic potential of these disparate regions. Our results suggest that distributed IED coupling between node communities is largely driven by outflow or propagation of IEDs from regions most strongly overlapping the SOZ. These findings are compatible with those of Diamond *et al*.,^34^ who utilized a similar network-based methodology to extract IED communities and quantify transmission latencies between SOZ and NSOZ regions. As their study included only adults, our results represent an encouraging replication of their core finding in the paediatric population. Considering the relative abundance of IEDs compared with seizures, reliable automated delineation of the IZ using co-activation latency as a biomarker may allow for more efficient invasive monitoring and shorter epilepsy monitoring unit stays. This alone would represent a significant advance in clinical practice. In terms of therapeutic potential, established modalities like responsive neuro-stimulation and deep brain stimulation interface with the epileptic brain at the level of invasive macro-electrodes. Directly intervening upon pathologic, hypersynchronous activity at the scale of cortical and subcortical networks has never been more feasible. Better understanding how brain regions synchronize during IEDs may allow us to identify time periods of heightened seizure vulnerability, and characterizing how IEDs flow through the network may help determine which network nodes to stimulate to drive the brain towards a less excitable state. In this regard, the observation that IED synchrony tends to increase in the hour preceding seizures is especially intriguing as a potential indicator of heightened seizure risk. We speculate that integrating a time-varying measure of IED network efficiency into seizure forecasting algorithms could improve their reliability, if generalizability can be confirmed through appropriate prospective validation.

From a pathophysiological perspective, the results from this study support a model of the irritative cortex as a network organized around a set of core node communities. The strength of IED synchrony between communities, as well as the relative activity rates of the communities, may vary from one time window to the next, but no patient exhibited a dramatic re-distribution of community membership over time, suggesting that these are stable features of the epileptic network. The tendency for IEDs to propagate through local communities in stereotyped fashion has been described elsewhere,^29,37^ including in previous work from our group.^31^ We postulate that these repeated local spike trains reflect the existence of an entrained, low-resistance circuit allowing spikes to flow directionally from upstream IED generators to downstream, functionally connected tissues. This observation raises questions about the mechanism of propagated IEDs, which may occur thousands or even hundreds of thousands of times per day in a given community. One hypothesis is that repeated IEDs directly shape the regional wiring of the epileptogenic network through plasticity-dependent mechanisms. A second is that altered developmental instructions induce abnormal connections that generate spatiotemporally stereotyped regional spike trains. Either way, it is intuitive to question whether networks that enable efficient IED synchronization are implicated in seizure generation and propagation.

Time-varying synchrony of IEDs between communities has been explored previously, mainly in the context of arousal and sleep–wake cycles.^15,34^ We hypothesized that synchronization of IEDs between communities requires the breakdown of inhibitory mechanisms that otherwise keep IED communities functionally isolated from one another. Intriguing parallels may be drawn to the so-called ‘interictal suppression hypothesis’, wherein SOZ nodes are functionally isolated by a distributed inhibitory network during the interictal state,^53^ which presumably breaks down in advance of seizures. To test this hypothesis, we explored two potential mechanisms underlying IED synchrony between communities. First, during periods of more diffuse IED network synchrony, we found increased oscillatory coherence between communities. Coordination in the spectral domain may align disparate neural populations for synchronized activity, and previous work has postulated such changes in spectral coherence in advance of propagated IEDs.^54^ Second, we revealed a reduction in measures of the post-spike SW during periods of diffuse IED synchrony, which may reflect a diminishment or ‘surrender’ of suppressive inhibitory tone either from regional circuitry (e.g. inhibitory innervation from local interneurons) or from more distant locations (e.g. thalamus) that otherwise serve to prevent IEDs from becoming synchronized between communities.^48^ The novel insights derived from this study generate many interesting hypotheses for further basic and clinical research in both animal models and humans.

We acknowledge several limitations of our study. First, as with all research involving invasive EEG, spatial sampling is inherently restricted to the recording array, and the 200 Hz sampling rate may be insensitive to subtle lags between IEDs or differences in waveform morphology. Second, while small cohort sizes are expected for studies of this kind, our analysis only includes 20 patients. Expansion of this cohort to include patients from multiple institutions, as well as adult patients, should be pursued to assess generalizability. Analysis of IED waveform morphology at the level of macro-electrode contacts as a surrogate for local excitatory or inhibitory tone is limited by the methodology. Deeper investigation into local electrophysiological changes in the epileptic cortex would require more refined techniques such as micro-electrode arrays to sample local field potentials and unit activity. Finally, automated spike detection is imperfect, although we have validated ours against human performance in prior studies.^36^

In conclusion, we have characterized IED co-activation as a marker of pathologic network synchrony across the epileptic cortex in children with refractory seizures. Our results describe a model of the epileptic network based on highly cohesive node communities with stereotyped local activation patterns, whose dynamic synchronization varies with factors such as seizure proximity, coherence and excitatory/inhibitory balance. The study sheds new light on the complex relationship between IEDs and seizures, improves our understanding of IEDs in the work-up for epilepsy surgery and steers us towards promising translational research in the domain of responsive neuro-modulation.

## Supplementary Material

fcaf127_Supplementary_Data

## Data Availability

Core scripts and sample data are available online (https://github.com/samuelbtomlinson/IED-Coactivation-Public) at the time of publication.
