# Supplementary material for: Co-activation of interictal epileptiform discharges localizes seizure onset zone and fluctuates with brain state
Source: Brain Commun. 2025 Apr 3;7(2):fcaf127. doi: 10.1093/braincomms/fcaf127 (PMC11982441; doi:10.1093/braincomms/fcaf127)
Supplement: fcaf127_Supplementary_Data [file fcaf127_supplementary_data.pdf]

Supplementary Materials

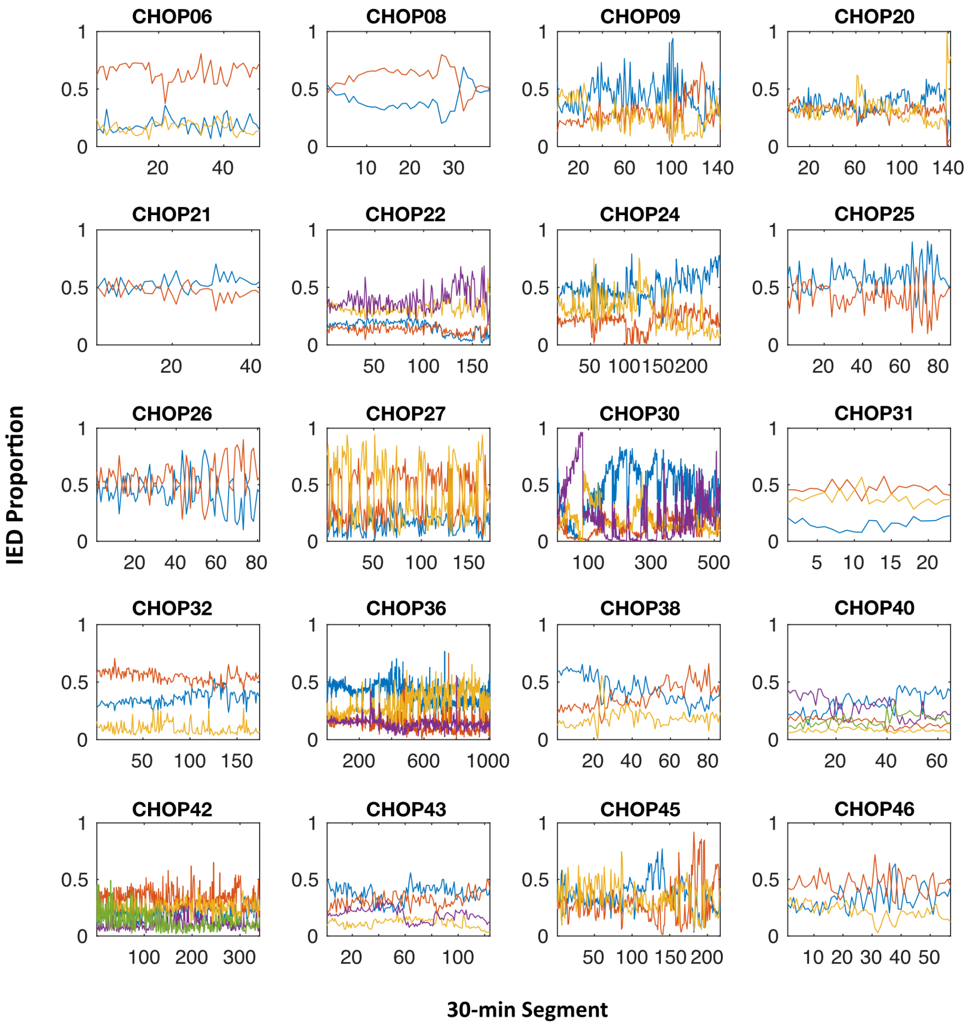

Supplementary Figure 1. For each patient, the proportion of total IEDs accounted for by each IED community (color-coded) is shown across segments. Tremendous temporal variability was observed in some patients, whereas others (e.g., CHOP31) demonstrated a stable distribution of IEDs across communities over time. *Abbreviations- IED: interictal epileptiform discharge.*

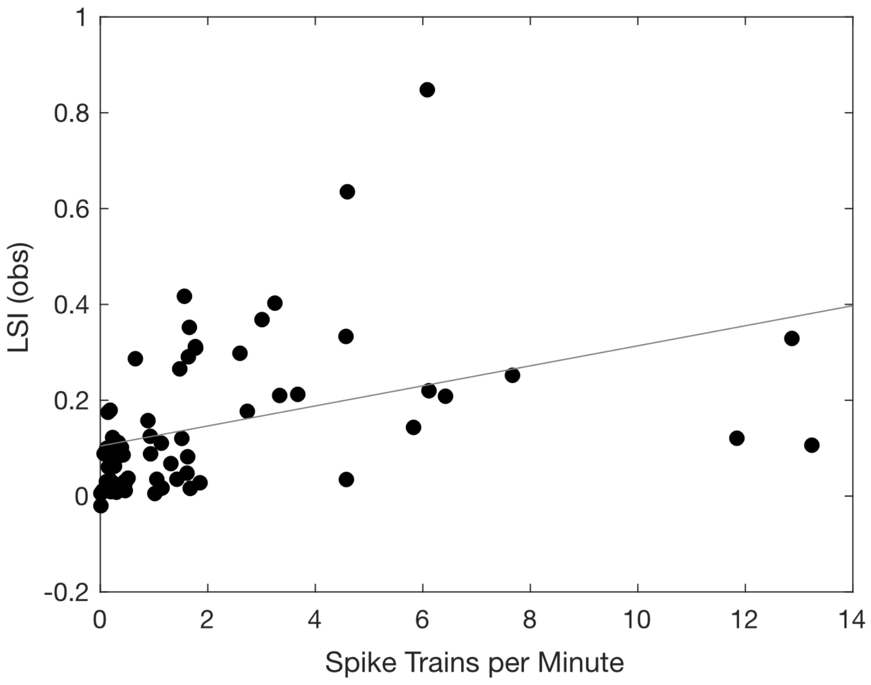

Supplementary Figure 2. Communities with a higher rate of spike train activity (spike trains/minute) exhibited more consistent spike train latencies (LSI_{obs}) (Spearman's r=0.58, p<0.001). Each data point represents a node community (n=64). *Abbreviations- LSI: Latency Similarity Index.*

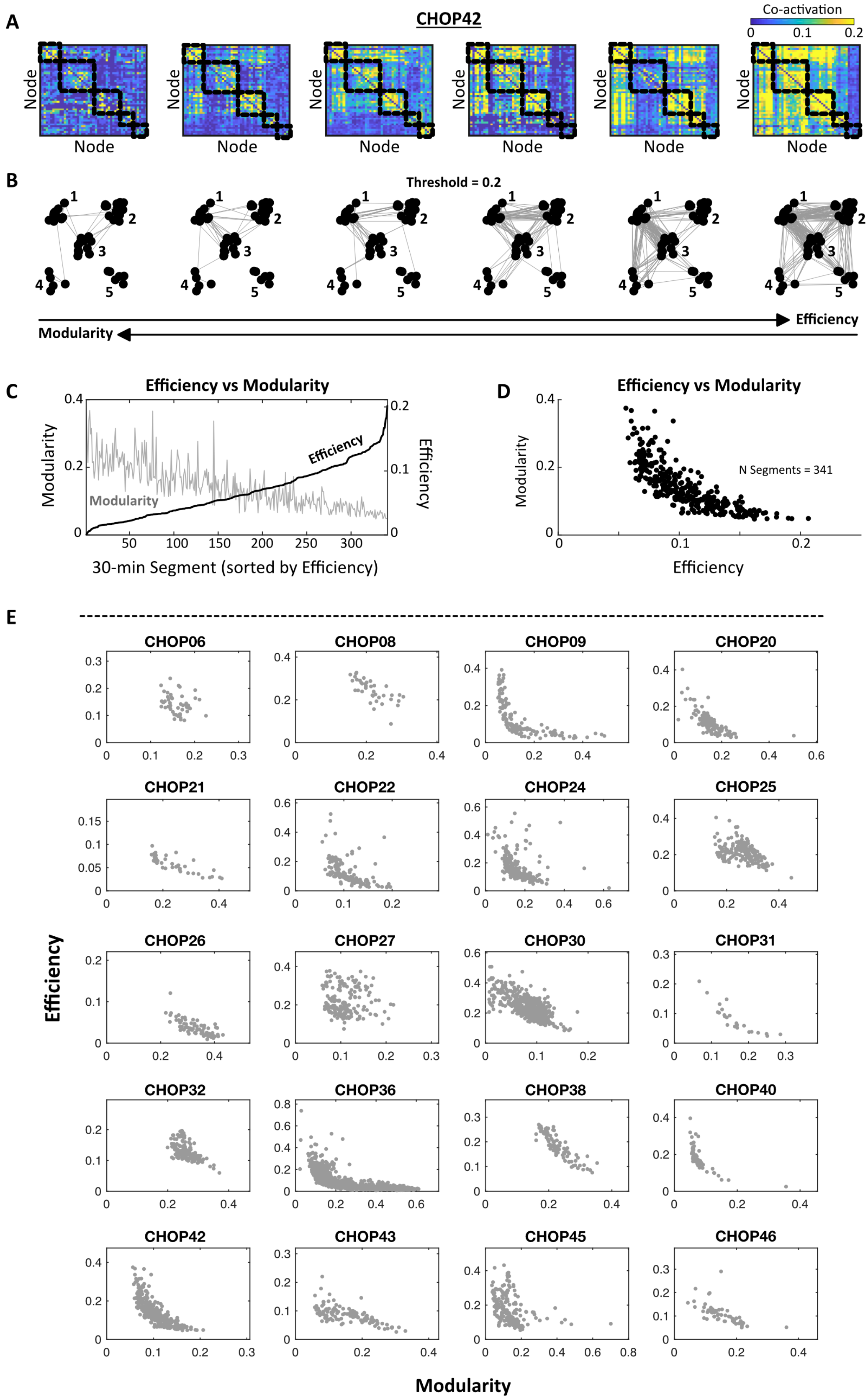
A
CHOP42
Co-activation
0
0.1
0.2
Node
Node
B
Threshold = 0.2
1
2
3
4
5
Modularity
Efficiency
C
Efficiency vs Modularity
Modularity
Efficiency
30-min Segment (sorted by Efficiency)
D
Efficiency vs Modularity
N Segments = 341
Modularity
Efficiency
E
CHOP06
CHOP08
CHOP09
CHOP20
CHOP21
CHOP22
CHOP24
CHOP25
CHOP26
CHOP27
CHOP30
CHOP31
CHOP32
CHOP36
CHOP38
CHOP40
CHOP42
CHOP43
CHOP45
CHOP46
Efficiency
Modularity

Supplementary Figure 3. Relationship between IED network efficiency and modularity. (A) Six representative IED co-activation matrices for CHOP42, each encoding IED co-activation from a 30-minute segment. This patient exhibited five IED node communities (dotted boxes). (B) Efficiency is a topological network parameter reflecting the average distance between node pairs, where distance is inversely proportional to IED co-activation strength. Modularity is a related network measure encoding how readily network nodes can be segregated into distinct clusters. Here, co-activation matrices from (A) are represented abstractly as networks with five discrete node communities (1-5). Network connections are shown as gray lines. For visualization purposes, connections exceeding an IED co-activation threshold of 0.2 are shown. Networks are sorted by increasing efficiency and decreasing modularity. (C-D) Inverse relationship between efficiency and modularity is demonstrated across segments (n=341). (E) Relationship between efficiency and modularity for each individual patient in the study. Data points represent individual segments. *Abbreviations- IED: interictal epileptiform discharge.*

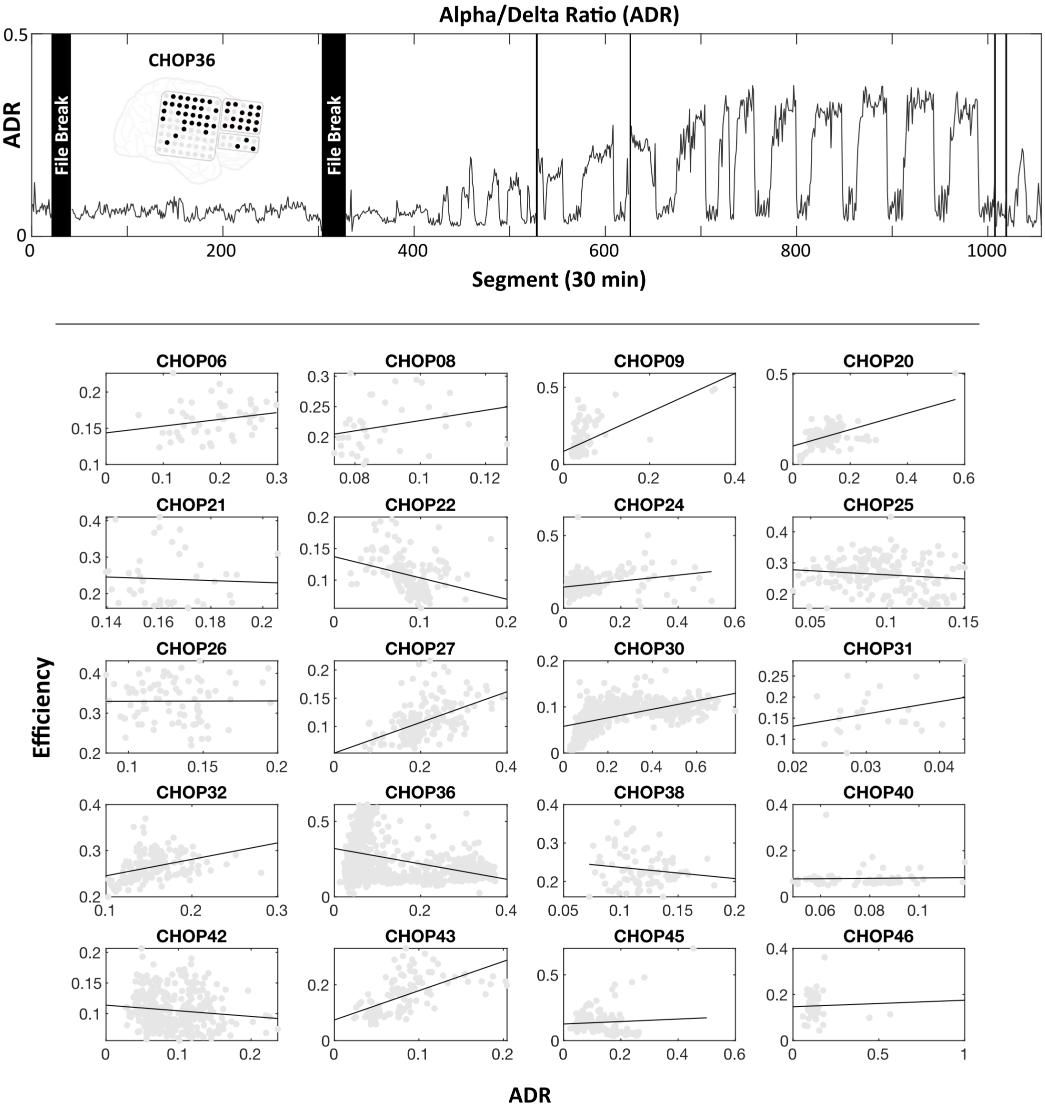

Supplementary Figure 4. Relationship between alpha-delta ratio (ADR) and network efficiency. *Top*: ADR calculation for representative patient (CHOP36). ADR was calculated in each segment from 60 randomly-selected 5-second epochs (5 minutes per segment) as the ratio of total power in the alpha (8-13 Hz) and delta (0.2-4 Hz) frequency bands. ADR is generally interpreted to distinguish periods of sleepfulness (low ADR) versus wakefulness (high ADR). *Bottom*: Correlation between ADR and network efficiency at the individual-patient level was highly non-uniform (markers = segments, line = least-squares fit). *Abbreviations- ADR: alpha delta ratio.*

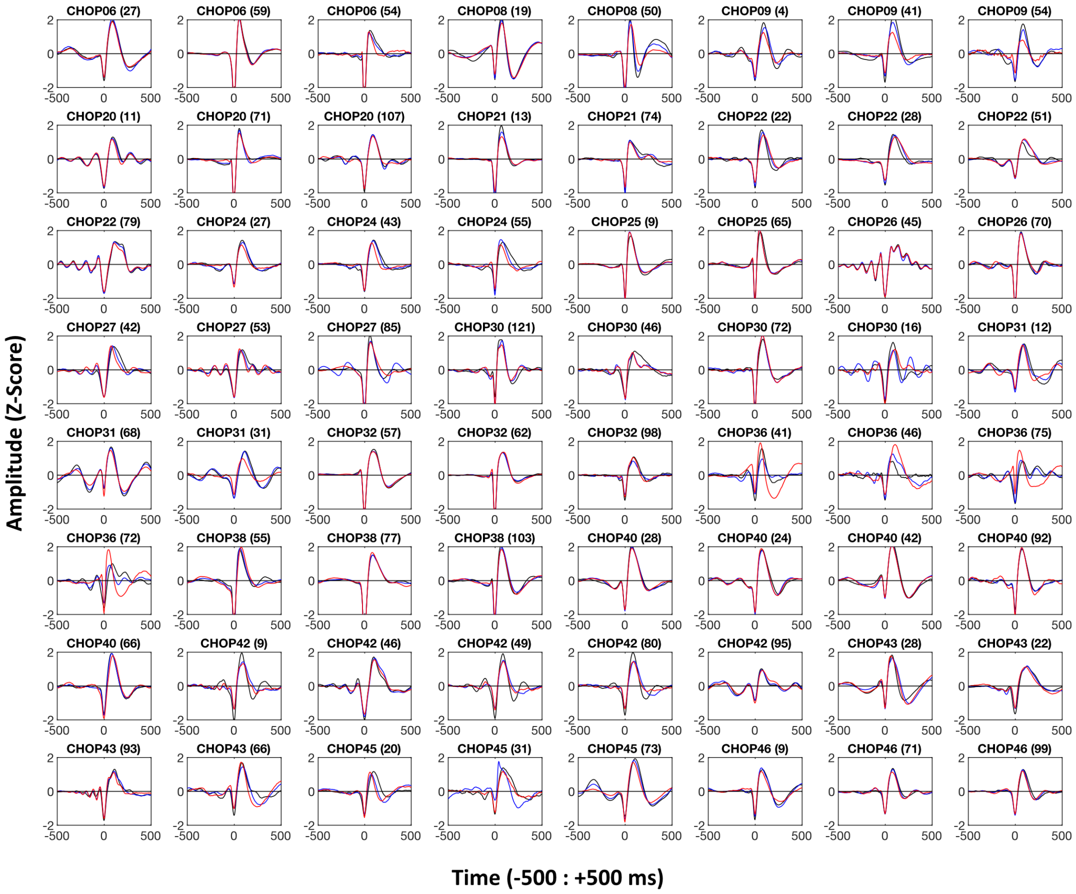

Supplementary Figure 5. IED waveform morphology. Grand-averaged IED waveforms are presented for all communities (n=64) examined in the assessment of post-spike slow wave (SW) morphology. Each plot depicts the grand-averaged normalized waveform (z-score) for each efficiency state (red: High efficiency, gray: Intermediate efficiency, blue: Low efficiency) over 1-second window centered around the IED peak. Plot titles show the patient and the corresponding electrode (parentheses). *Abbreviations- IED: interictal epileptiform discharge.*

Supplemental Table 1 - Local Community Activity

ID	Community	Size (#Nodes)	SOZ Overlap (%)	IED Density (IEDs/ node/min)	Cohesion	Community Latency (ms)	LSI Observed	LSI Surrogate (Median, 95% CI)
**CHOP06**	1	8	12.50	4.13	1.00	-8.04	0.027 (N.S.)	0.002 (-0.049-0.048)
	2	10	40.00	10.34	1.00	16.08	0.848	-0.002 (-0.030-0.033)
	3	6	16.67	4.77	0.65	-8.61	0.352	-0.007 (-0.068-0.047)
**CHOP08**	1	18	0.00	4.74	1.00	8.79	0.309	0.000 (-0.018-0.015)
	2	20	65.00	6.44	1.00	-8.79	0.417	-0.003 (-0.022-0.023)
**CHOP09**	1	17	29.41	2.40	1.00	-2.39	0.006 (N.S.)	0.001 (-0.029-0.026)
	2	9	44.44	3.20	1.00	-5.71	0.005 (N.S.)	-0.007 (-0.039-0.034)
	3	12	41.67	2.97	0.99	7.26	0.017 (N.S.)	0.002 (-0.031-0.029)
**CHOP20**	1	17	0.00	8.11	1.00	-7.76	0.112	-0.001 (-0.019-0.019)
	2	12	50.00	9.56	0.92	3.31	0.635	-0.001 (-0.026-0.027)
	3	14	21.43	7.64	0.81	4.50	0.079	0.006 (-0.024-0.030)
**CHOP21**	1	27	44.44	10.65	1.00	22.65	0.035	-0.002 (-0.018-0.018)
	2	30	3.33	7.83	1.00	-23.26	0.082	0.000 (-0.014-0.016)
**CHOP22**	1	7	42.86	3.84	0.56	0.89	0.030 (N.S.)	0.002 (-0.065-0.042)
	2	6	16.67	3.78	0.89	1.88	0.048 (N.S.)	-0.011 (-0.047-0.053)
	3	14	0.00	3.83	1.00	1.87	0.034 (N.S.)	-0.001 (-0.033-0.024)
	4	14	14.29	4.60	0.86	-3.03	0.011 (N.S.)	0.000 (-0.024-0.022)
**CHOP24**	1	13	53.85	2.69	0.79	6.10	0.101	0.005 (-0.020-0.030)
	2	6	16.67	2.55	0.69	-3.57	0.016 (N.S.)	0.004 (-0.048-0.053)
	3	10	20.00	2.18	0.93	-4.19	0.266	0.000 (-0.031-0.039)
**CHOP25**	1	24	0.00	12.38	1.00	-9.58	0.210	-0.000 (-0.016-0.017)
	2	19	63.16	16.31	1.00	9.58	0.106	0.002 (-0.016-0.018)
**CHOP26**	1	21	0.00	6.46	1.00	-5.19	-0.02 (N.S.)	-0.001 (-0.021-0.033)
	2	21	80.95	8.85	1.00	5.19	0.212	-0.002 (-0.020-0.015)
**CHOP27**	1	10	0.00	5.54	0.97	-0.25	0.068	-0.003 (-0.029-0.040)
	2	20	0.00	6.80	1.00	-1.83	0.158	0.001 (-0.024-0.021)
	3	19	36.84	8.63	0.99	2.36	0.097	0.002 (-0.017-0.017)
**CHOP30**	1	18	0.00	5.00	0.82	0.05	0.062	-0.004 (-0.019-0.032)
	2	9	22.22	2.98	0.84	0.60	0.122	0.003 (-0.024-0.033)
	3	9	0.00	3.70	0.79	0.25	0.061	-0.008 (-0.037-0.048)
	4	10	0.00	3.06	0.81	-0.96	0.100	0.002 (-0.040-0.032)
**CHOP31**	1	7	57.14	7.53	1.00	14.16	0.208	-0.001 (-0.027-0.033)
	2	14	85.71	9.15	1.00	-3.50	0.143	-0.001 (-0.027-0.025)
	3	15	33.33	6.65	1.00	-5.24	0.175	0.003 (-0.025-0.037)
**CHOP32**	1	8	0.00	21.87	1.00	-5.04	0.121	0.008 (-0.035-0.039)
	2	13	53.85	21.15	1.00	6.81	0.329	-0.001 (-0.023-0.025)
	3	6	0.00	7.84	0.68	-6.97	0.333	0.007 (-0.049-0.059)
**CHOP36**	1	19	42.11	3.03	0.71	5.29	0.291	-0.002 (-0.018-0.009)
	2	9	0.00	2.26	0.78	-21.57	0.287	-0.004 (-0.023-0.022)
	3	14	7.14	2.93	0.96	-2.13	0.125	0.001 (-0.017-0.021)
	4	7	0.00	2.79	0.88	2.12	0.312	0.003 (-0.025-0.022)
**CHOP38**	1	20	0.00	8.53	0.99	-10.96	0.403	-0.001 (-0.024-0.014)
	2	15	60.00	10.03	1.00	14.19	0.252	-0.002 (-0.018-0.025)
	3	9	11.11	8.80	1.00	-4.23	0.220	-0.005 (-0.036-0.025)
**CHOP40**	1	16	100.00 (Generalized)	5.02	1.00	-9.11	0.086	0.003 (-0.029-0.027)
	2	8	100.00 (Generalized)	5.26	0.72	7.23	0.035 (N.S.)	0.001 (-0.043-0.035)
	3	5	100.00 (Generalized)	4.42	1.00	-3.62	0.120	-0.000 (-0.050-0.042)
	4	17	100.00 (Generalized)	4.72	1.00	3.14	0.179	-0.003 (-0.034-0.020)
	5	7	100.00 (Generalized)	5.23	1.00	0.15	0.110	0.004 (-0.020-0.047)
**CHOP42**	1	11	36.36	2.96	0.65	-2.01	0.031 (N.S.)	-0.005 (-0.029-0.032)
	2	15	26.67	3.29	1.00	3.35	0.088	0.001 (-0.024-0.024)
	3	12	33.33	3.49	0.88	-1.46	0.037 (N.S.)	0.004 (-0.021-0.034)
	4	5	0.00	3.09	0.72	-1.72	0.011 (N.S.)	-0.006 (-0.035-0.044)
	5	6	0.00	2.88	0.99	0.53	0.027 (N.S.)	0.009 (-0.055-0.078)
**CHOP43**	1	11	81.82	13.80	0.89	17.62	0.368	0.001 (-0.036-0.033)
	2	19	10.53	6.81	0.99	-17.68	0.088	0.001 (-0.025-0.017)
	3	7	0.00	6.47	0.69	-2.47	0.022 (N.S.)	0.003 (-0.039-0.046)
	4	11	81.82	7.71	0.85	11.87	0.298	-0.003 (-0.036-0.026)
**CHOP45**	1	15	6.67	5.85	0.76	-0.35	0.017 (N.S.)	-0.001 (-0.027-0.025)
	2	15	0.00	5.22	0.86	-4.08	0.008 (N.S.)	-0.002 (-0.027-0.031)
	3	11	54.55	6.56	0.76	10.50	0.035 (N.S.)	-0.002 (-0.029-0.037)
**CHOP46**	1	17	35.29	12.58	0.93	3.50	0.177	0.001 (-0.017-0.017)
	2	28	0.00	12.38	0.91	-1.49	0.009 (N.S.)	0.001 (-0.018-0.017)
	3	14	0.00	11.55	1.00	-1.18	0.028 (N.S.)	-0.003 (-0.024-0.020)

Supplemental Table 2 - Diminishment of Post-Spike Slow Wave

			IEDs (N)				SW Duration (ms)			SW Amplitude (Z-score)			SW AUC		
ID	Community	Electrode	High Eff	Int Eff	Low Eff	Analyzed	High Eff (REF)	Int Eff (%Change)	Low Eff (%Change)	High Eff (REF)	Int Eff (%Change)	Low Eff (%Change)	High Eff (REF)	Int Eff (%Change)	Low Eff (%Change)
**CHOP06**	1	27	1433	757	1191	757	270	280 (1.82)	270 (0.00)	3.28	3.47 (2.83)	3.53 (3.59)	49.43	58.71 (8.58)	53.63 (4.08)
	2	59	3999	3516	3046	3046	190	195 (1.30)	205 (3.80)	5.20	5.26 (0.50)	5.49 (2.64)	38.82	38.83 (0.02)	42.59 (4.64)
	3	54	1576	940	615	615	155	240 (21.52)	265 (26.19)	3.91	3.62 (-3.85)	3.77 (-1.78)	24.88	31.22 (11.29)	38.58 (21.58)
**CHOP08**	1	19	1920	1354	841	841	205	200 (-1.23)	195 (-2.50)	3.05	3.46 (6.29)	3.72 (9.84)	42.38	44.72 (2.68)	46.61 (4.75)
	2	50	1915	2257	2834	1915	160	140 (-6.67)	135 (-8.47)	3.70	4.21 (6.44)	4.60 (10.77)	30.59	31.67 (1.74)	33.87 (5.09)
**CHOP09**	1	4	652	333	296	296	220	255 (7.37)	230 (2.22)	2.61	3.07 (8.13)	3.43 (13.61)	28.97	40.43 (16.51)	42.97 (19.47)
	2	41	1056	782	1866	782	235	255 (4.08)	265 (6.00)	2.38	3.17 (14.12)	4.03 (25.69)	28.70	45.53 (22.67)	54.59 (31.08)
	3	54	785	553	109	109	170	230 (15.00)	240 (17.07)	1.96	2.88 (19.03)	3.41 (26.99)	18.84	35.84 (31.08)	42.11 (38.18)
**CHOP20**	1	11	2535	2012	1054	1054	185	200 (3.90)	205 (5.13)	2.74	2.95 (3.65)	3.03 (5.05)	24.96	28.73 (7.02)	31.70 (11.88)
	2	71	3406	2712	1709	1709	210	185 (-6.33)	200 (-2.44)	4.22	4.30 (0.95)	4.49 (3.03)	30.49	32.51 (3.20)	34.71 (6.47)
	3	107	2455	2028	1103	1103	235	215 (-4.44)	220 (-3.30)	3.12	3.22 (1.50)	3.36 (3.72)	32.20	35.12 (4.33)	35.39 (4.72)
**CHOP21**	1	13	5983	3224	2634	2634	215	220 (1.15)	205 (-2.38)	3.21	3.98 (10.74)	4.54 (17.19)	30.52	36.54 (8.98)	39.45 (12.76)
	2	74	6470	2910	1766	1766	180	270 (20.00)	225 (11.11)	2.68	3.16 (8.19)	3.00 (5.58)	19.89	27.09 (15.32)	29.69 (19.75)
**CHOP22**	1	22	1104	773	975	773	260	245 (-2.97)	230 (-6.12)	2.85	3.18 (5.58)	3.41 (8.96)	37.66	38.33 (0.88)	39.67 (2.60)
	2	28	1175	889	899	889	355	290 (-10.08)	260 (-15.45)	2.61	2.85 (4.39)	3.00 (6.89)	43.25	41.81 (-1.68)	36.58 (-8.35)
	3	51	982	1039	1594	982	320	305 (-2.40)	295 (-4.07)	2.30	2.29 (-0.30)	2.02 (-6.54)	36.97	34.69 (-3.17)	24.37 (-20.53)
	4	79	1346	2482	3171	1346	280	185 (-20.43)	190 (-19.15)	2.86	3.02 (2.58)	3.02 (2.58)	41.78	36.45 (-6.81)	38.49 (-4.09)
**CHOP24**	1	27	985	831	385	385	230	300 (13.21)	300 (13.21)	2.52	2.45 (-1.38)	2.58 (1.22)	24.94	36.12 (18.31)	37.84 (20.56)
	2	43	988	638	430	430	205	255 (10.87)	305 (19.61)	2.80	2.97 (3.03)	3.05 (4.26)	25.73	34.54 (14.63)	40.64 (22.47)
	3	55	771	511	355	355	230	230 (0.00)	310 (14.81)	2.66	3.26 (10.22)	2.88 (4.05)	25.74	33.34 (12.85)	39.93 (21.60)
**CHOP25**	1	9	6613	4554	2972	2972	210	220 (2.33)	205 (-1.20)	3.94	4.10 (2.07)	3.65 (-3.74)	36.20	37.80 (2.17)	35.62 (-0.80)
	2	65	8863	6312	1948	1948	200	200 (0.00)	215 (3.61)	4.35	4.41 (0.67)	3.94 (-4.99)	35.75	35.52 (-0.32)	38.13 (3.23)
**CHOP26**	1	45	1686	1784	1514	1514	140	140 (0.00)	140 (0.00)	2.98	2.95 (-0.50)	2.99 (0.20)	25.71	24.86 (-1.67)	26.27 (1.07)
	2	70	3204	2569	1475	1475	240	230 (-2.13)	220 (-4.35)	4.61	4.61 (0.00)	4.53 (-0.88)	40.47	41.45 (1.20)	40.44 (-0.03)
**CHOP27**	1	42	2473	990	331	331	240	260 (4.00)	275 (6.80)	3.01	3.00 (-0.05)	2.97 (-0.56)	29.69	35.90 (9.47)	41.57 (16.67)
	2	53	3365	2069	846	846	150	160 (3.23)	190 (11.76)	2.89	2.84 (-0.78)	2.81 (-1.29)	22.33	24.43 (4.48)	30.01 (14.68)
	3	85	3469	3531	4070	3469	255	240 (-3.03)	205 (-10.87)	4.32	4.00 (-3.84)	5.04 (7.74)	38.51	40.51 (2.53)	39.89 (1.75)
**CHOP30**	1	121	3519	2304	824	824	170	190 (5.56)	185 (4.23)	3.62	3.91 (3.88)	3.38 (-3.38)	31.15	36.37 (7.72)	37.69 (9.49)
	2	46	1074	1180	454	454	175	200 (6.67)	410 (40.17)	2.70	2.87 (2.96)	2.71 (0.08)	21.65	22.28 (1.45)	44.10 (34.15)
	3	72	2709	1475	1697	1475	235	235 (0.00)	245 (2.08)	4.81	4.53 (-3.02)	4.06 (-8.40)	48.98	47.19 (-1.85)	43.28 (-6.18)
	4	16	185	904	1478	185	255	180 (-17.24)	190 (-14.61)	3.19	2.97 (-3.57)	3.41 (3.31)	31.79	26.68 (-8.74)	33.46 (2.56)
**CHOP31**	1	12	3556	1227	464	464	210	235 (5.62)	290 (16.00)	2.56	2.85 (5.37)	2.67 (2.23)	29.84	34.01 (6.52)	43.37 (18.48)
	2	68	4570	2512	1085	1085	215	215 (0.00)	215 (0.00)	2.72	2.38 (-6.61)	2.45 (-5.21)	35.60	38.12 (3.41)	41.01 (7.06)
	3	31	2893	1556	639	639	280	300 (3.45)	310 (5.08)	2.17	2.69 (10.70)	2.79 (12.40)	26.96	41.12 (20.80)	45.54 (25.64)
**CHOP32**	1	57	7930	8844	7714	7714	260	260 (0.00)	260 (0.00)	3.66	3.66 (-0.01)	3.91 (3.33)	44.30	43.80 (-0.56)	47.42 (3.41)
	2	62	8501	6679	5473	5473	255	240 (-3.03)	240 (-3.03)	4.25	4.09 (-2.02)	4.08 (-2.12)	42.98	35.46 (-9.59)	36.87 (-7.65)
	3	98	1344	871	696	696	210	80 (-44.83)	230 (4.55)	2.33	1.69 (-15.93)	2.57 (4.84)	24.81	9.75 (-43.60)	26.83 (3.90)
**CHOP36**	1	41	1609	495	301	301	200	180 (-5.26)	190 (-2.56)	2.96	2.05 (-18.13)	3.08 (2.12)	42.98	17.12 (-43.02)	29.60 (-18.44)
	2	46	417	107	300	107	115	365 (52.08)	80 (-17.95)	3.02	2.65 (-6.64)	2.49 (-9.64)	24.65	42.93 (27.06)	12.58 (-32.42)
	3	75	842	585	769	585	180	160 (-5.88)	235 (13.25)	2.59	2.51 (-1.54)	2.51 (-1.61)	26.22	17.51 (-19.93)	22.85 (-6.86)
	4	72	1050	447	681	447	185	150 (-10.45)	220 (8.64)	3.71	2.23 (-24.81)	2.58 (-18.03)	36.31	16.09 (-38.59)	26.47 (-15.67)
**CHOP38**	1	55	2897	1213	2423	1213	260	240 (-4.00)	225 (-7.22)	4.24	4.73 (5.40)	5.69 (14.55)	45.74	42.04 (-4.22)	42.79 (-3.33)
	2	77	4440	2182	1993	1993	315	320 (0.79)	295 (-3.28)	5.13	4.93 (-2.02)	4.94 (-1.89)	55.01	52.85 (-2.01)	51.96 (-2.85)
	3	103	3272	1977	1449	1449	240	240 (0.00)	225 (-3.23)	3.99	4.45 (5.41)	4.62 (7.22)	46.91	49.55 (2.73)	49.19 (2.37)
**CHOP40**	1	28	1931	1382	914	914	250	255 (0.99)	270 (3.85)	3.65	3.80 (2.13)	3.81 (2.26)	46.84	48.67 (1.92)	51.48 (4.73)
	2	24	1807	1124	514	514	235	230 (-1.08)	250 (3.09)	3.82	3.69 (-1.71)	3.72 (-1.35)	46.18	43.55 (-2.93)	47.15 (1.04)
	3	42	1203	827	443	443	245	245 (0.00)	260 (2.97)	3.09	3.13 (0.57)	3.17 (1.25)	50.64	50.05 (-0.59)	55.60 (4.67)
	4	92	1600	800	389	389	200	210 (2.44)	220 (4.76)	4.00	3.91 (-1.11)	3.56 (-5.78)	41.21	40.94 (-0.32)	40.78 (-0.52)
	5	66	1108	993	752	752	250	235 (-3.09)	235 (-3.09)	3.69	3.60 (-1.34)	3.58 (-1.62)	45.14	44.56 (-0.65)	45.19 (0.06)
**CHOP42**	1	9	803	705	281	281	240	290 (9.43)	200 (-9.09)	2.65	2.86 (3.86)	4.06 (21.11)	31.87	38.62 (9.58)	40.86 (12.37)
	2	46	1033	758	654	654	305	325 (3.17)	290 (-2.52)	3.21	3.49 (4.10)	3.70 (7.06)	46.74	54.80 (7.94)	46.61 (-0.14)
	3	49	874	827	273	273	220	295 (14.56)	200 (-4.76)	2.87	2.86 (-0.16)	3.82 (14.12)	34.00	39.10 (6.98)	41.35 (9.75)
	4	80	847	574	393	393	245	260 (2.97)	220 (-5.38)	2.88	2.74 (-2.46)	3.71 (12.63)	33.89	36.21 (3.32)	44.45 (13.48)
	5	95	782	708	970	708	195	195 (0.00)	205 (2.50)	1.89	1.80 (-2.66)	1.87 (-0.57)	21.40	18.86 (-6.31)	18.35 (-7.67)
**CHOP43**	1	28	8596	5305	4592	4592	240	255 (3.03)	210 (-6.67)	3.09	3.01 (-1.20)	2.99 (-1.66)	46.47	47.37 (0.96)	38.92 (-8.84)
	2	22	2692	1684	2089	1684	390	415 (3.11)	385 (-0.65)	2.47	2.53 (1.24)	2.81 (6.46)	41.78	44.45 (3.10)	46.31 (5.15)
	3	93	2080	2000	1546	1546	100	290 (48.72)	270 (45.95)	2.47	2.75 (5.42)	3.03 (10.21)	17.07	33.25 (32.15)	33.55 (32.55)
	4	66	4096	1566	323	323	265	285 (3.64)	205 (-12.77)	2.77	2.49 (-5.35)	3.11 (5.75)	42.38	39.71 (-3.24)	33.56 (-11.61)
**CHOP45**	1	20	2262	515	895	515	185	205 (5.13)	240 (12.94)	2.62	2.40 (-4.50)	2.55 (-1.40)	22.29	24.18 (4.07)	29.48 (13.89)
	2	31	767	347	280	280	250	245 (-1.01)	265 (2.91)	2.49	2.25 (-4.96)	2.74 (4.77)	31.26	41.26 (13.78)	41.04 (13.53)
	3	73	3035	1964	1774	1774	280	290 (1.75)	295 (2.61)	3.48	3.48 (-0.09)	3.40 (-1.17)	46.75	53.36 (6.60)	57.83 (10.60)
**CHOP46**	1	9	5290	2909	2881	2881	265	250 (-2.91)	245 (-3.92)	2.51	2.86 (6.54)	2.96 (8.24)	31.96	39.29 (10.29)	37.23 (7.62)
	2	71	4735	4060	2042	2042	205	200 (-1.23)	220 (3.53)	2.45	2.61 (3.27)	2.69 (4.80)	26.57	27.15 (1.08)	29.41 (5.06)
	3	99	4342	3273	1477	1477	215	225 (2.27)	240 (5.49)	2.53	2.62 (1.88)	2.61 (1.64)	26.76	29.55 (4.94)	31.44 (8.04)
